# Supplementary material for: Predicting visual function by interpreting a neuronal wiring diagram
Source: Nature. 2024 Oct 2;634(8032):113–23. doi: 10.1038/s41586-024-07953-5 (PMC11446822; doi:10.1038/s41586-024-07953-5)
Supplement: Supplementary file 6 — CRF and ERF predictions for individual TmY4 and TmY9 cells. Analogous to Supplementary Data 3, but for TmY target types. Shown are the top four monosynaptic pathways, the strongest pathway passing through each of the top ten intermediary types (ranking from Extended Data Fig. 7), and the trisynaptic pathway Tm1–TmY–Dm3–TmY (see the section entitled Prediction of spatial normalization). [file 41586_2024_7953_MOESM6_ESM.zip › DataS4/TmY9qΓèÑ/720575940624771255.pdf]

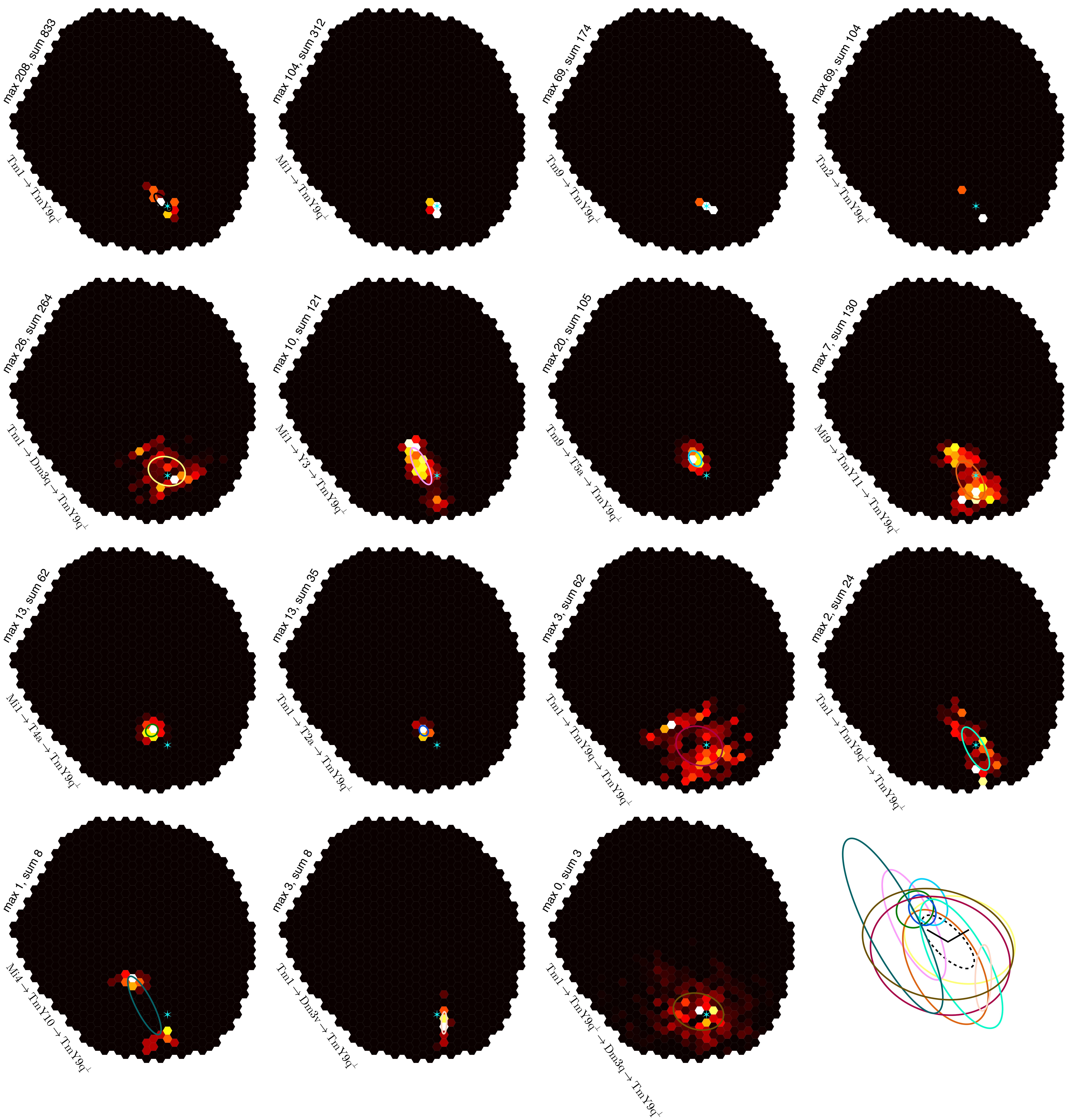
max 208, sum 833
Tm1 → TmY9q⊥
max 104, sum 312
Mi1 → TmY9q⊥
max 69, sum 174
Tm9 → TmY9q⊥
max 69, sum 104
Tm2 → TmY9q⊥
max 26, sum 264
Tm1 → Dm3q → TmY9q⊥
max 10, sum 121
Mi1 → Y3 → TmY9q⊥
max 20, sum 105
Tm9 → T5a → TmY9q⊥
max 7, sum 130
Mi9 → TmY11 → TmY9q⊥
max 13, sum 62
Mi1 → T4a → TmY9q⊥
max 13, sum 35
Tm1 → T2a → TmY9q⊥
max 3, sum 62
Tm1 → TmY9q → TmY9q⊥
max 2, sum 24
Tm1 → TmY9q⊥ → TmY9q⊥
max 1, sum 8
Mi4 → TmY10 → TmY9q⊥
max 3, sum 8
Tm1 → Dm3v → TmY9q⊥
max 0, sum 3
Tm1 → TmY9q⊥ → Dm3q → TmY9q⊥
